# Supplementary material for: A critical issue in model-based inference for studying trait-based community assembly and a solution
Source: PeerJ. 2017 Jan 12;5:e2885. doi: 10.7717/peerj.2885 (PMC5237366; doi:10.7717/peerj.2885)
Supplement: Appendix S1 — Mathematical description of the Gaussian response model and log-linear model used in the simulation. [file peerj-05-2885-s001.pdf]

A critical issue in model-based inference for studying trait-based community assembly and a solution

Cajo J. F. ter Braak, Pedro R. Peres-Neto and Stéphane Dray

Appendix S1

1. The simulation models

In both simulation models the abundance y_{ij} of species j with trait value t_j in community i with environmental value e_i is drawn from a negative-binomial distribution NBI(μ_{ij}, σ, ν) with mean parameter μ_{ij}, overdispersion parameter 1, giving variance function $\mu_{ij} + \mu_{ij}^2$.

1.1 The Gaussian response model

Since Swan (1970), many numerical ecologists have evaluated their numerical and statistical methods using the Gaussian response model, and so did Dray and Legendre (2008), ter Braak et al.(2012) and Peres Neto et al. (2016) in evaluating the fourth-corner correlation and the Community Weigthed Means approach. In our simulations, the expected abundance μ_{ij} of species j with trait value z_j^* in community i with environmental value x_i^* is

$$\mu_{ij} = h_j \exp[-\frac{\left(x_i^* - z_j^*\right)^2}{2\sigma_j^2}], \qquad \text{(A.1)}$$

where

- h_j is the maximum value of species j which is drawn from the uniform distribution on the interval [3,10], *i.e.* $h_j \sim U(3,10)$.
- σ_j is the tolerance of species j which is drawn from the uniform distribution on the interval [0,2], *i.e.* $\sigma_j \sim U(0,2)$.

In the simulations, $z_j^* \sim N(0,1)$, and $x_i^* \sim N(0,1)$, so the setting of σ_j gives at least for some species visible unimodal response to $\mathbf{x}^*$ in the data.

The observed trait value t_j and observed environmental value e_i are generated as a combination of the trait and environmental variable used to generate the expected abundance (z_j^* and x_i^*) and new, independent normal draws $z_j \sim N(0,1)$ and $x_i \sim N(0,1)$:

$$t_j = \rho_t z_j^* + \sqrt{(1-\rho_t^2)} z_j \text{ and } e_i = \rho_e x_i^* + \sqrt{(1-\rho_e^2)} x_i, \qquad \text{(A.2)}$$

so that ρ_t and ρ_e are correlations between the observed trait and observed environmental variable with the variables used to generate the expected abundance

values. In the 'trait random' case $\rho_t = 0$ and $\rho_e = 1$, in the 'environment random' case $\rho_e = 0$ and $\rho_t = 1$, and in the 'both random case' $\rho_t = 0$ and $\rho_e = 0$. The strength of the trait-environment association is in this model dependent on a number of parameters: ρ_e, ρ_t and the parameters $\{\sigma_j\}$; the association is absent if $\rho_t\rho_e = 0$.

In the 'trait random' case $\rho_t = 0$ and $\rho_e = 1$, so that t_j=z_j and $e_i = x_i^*$, making the Gaussian model in Equation (A.1) equivalent with Equation (1) in the main text.

The essential point in the data generation is that the observed trait (t_j) and the variable (z_j^*) used to generate the Gaussian model in Equation (A.1) are standard normals with correlation ρ_t, and a similarly for e_i and x_i^*. In the R script, $\{t_j\}$ and $\{e_i\}$ are generated first and then $\{x_i^*\}$ and $\{z_j^*\}$ by addition of 'noise' variables $z_j \sim N(0,1)$ and $x_i \sim N(0,1)$, using an equation similar to Equation A.2,

$$z_j^* = \rho_t t_j + \sqrt{(1-\rho_t^2)}z_j \text{ and } x_i^* = \rho_e e_i + \sqrt{(1-\rho_e^2)}x_i, \quad \text{(A.2)}$$

This set-up is easy to generalize to multi-trait observations without generalizing Equation A.1: t_j is obtained by generating q trait values from a multivariate normal distribution $N(\mathbf{0}, \Sigma)$, calculating a linear combination $\mathbf{c}$ and dividing by the standard deviation of the sum, $(\mathbf{c}^T\Sigma\mathbf{c})^{1/2}$. In the simulations, $\Sigma = \mathrm{I}_q$ and $\mathbf{c} = \mathbf{1}_q^T$ so that the linear combination is simply the sum.

1.2 The log-linear simulation model

The log-linear simulation model is a generalized linear mixed model with main effects and interactions. In this model the expected abundance μ_{ij} of species j with trait value t_j in community *i* with environmental value e_i is

$$\log(\mu_{ij}) = \mu_0 + R_i + C_j + b_{te}t_j e_i + b_{ze}z_j e_i + b_{tx}t_j x_i + b_{zx}^* z_j^* x_i^* + \epsilon_{ij}, \quad \text{(A.3)}$$

where

- μ_0 is a parameter (intercept),
- R_i is the row main effect that is a function of *i* and/or e_i only,
- C_j is the column main effect that is a function of j and/or t_j only,
- $b_{te}, b_{ze}, b_{tx}, b_{zx}^*$ are parameters that govern the importance of the associated interaction terms,
- $z_j \sim N(0,1)$ and $z_j^* \sim N(0,1)$, two independent normal random variables, that could represent two unobserved trait variables that are independent of the observed variable t_j.
- $x_i \sim N(0,1)$ and $x_i^* \sim N(0,1)$, two independent normal random variables, that could represent two unobserved environmental variables that are independent of the observed variable e_i.
- $\epsilon_{ij} \sim N(0, \sigma_\epsilon^2)$, an independent normal random variable (with variance σ_ϵ^2) that could represent unobserved full rank variation leading to overdispersion in the

abundance beyond the overdispersion via the conditional distribution of y_{ij} given μ_{ij}.

For the purpose of trait-environment association the key parameters are b_{te}, b_{ze}, b_{tx} and the most important distinction is whether $b_{te} = 0$ or $b_{te} \neq 0$. If $b_{te} = 0$, then the trait and the environment may influence abundance through the row and column effects R_i and C_j, but there is no trait-environment association. However, if $b_{te} \neq 0$ then there is trait-environment association. With the simulations, we investigated whether the statistical methods under investigation can measure the trait-environment association and whether the associated significance tests can detect it, in particular whether these tests are valid and useful to test the null hypothesis $H_0\colon b_{te} = 0$ versus the alternative hypothesis $H_1\colon b_{te} \neq 0$. For validity, the tests should control the type I error α, taken as $\alpha = 0.05$ in the main text, that is, they should lead to at most $100\,\alpha\%$ rejections of the null hypothesis if in fact $H_0\colon b_{te} = 0$ holds (closer to $100\,\alpha\%$ is better). For usefulness, they should have sufficient power, that is, they should lead to a large fraction of rejections under the alternative hypothesis (larger is better).

The null hypothesis is a composite null hypothesis and there are four important cases.

- Case 1 has parameters $(b_{te} = 0, b_{ze} = 0, b_{tx} = 0)$ so that neither *e* nor *t* have an interaction effect. This is the 'both random' case.
- Case 2 has parameters $(b_{te} = 0, b_{ze} \neq 0, b_{tx} = 0)$ so that the environmental variable has an interaction effect with an unobserved variable *z*, possibly an unobserved trait variable that is uncorrelated with *t*. This is the 'trait random' case.
- Case 3 has parameters $(b_{te} = 0, b_{ze} = 0, b_{tx} \neq 0)$ so that the trait variable has an interaction effect with an unobserved variable *x*, possibly an unobserved environmental variable that is uncorrelated with e. This is the 'environment random' case.
- Case 4 has parameters $(b_{te} = 0, b_{ze} \neq 0, b_{tx} \neq 0)$ so that neither *e* nor *t* have an interaction effect. This case is not covered in the Gaussian simulation model. This is the 'both random interaction' case.

Case 1 is the simplest case and many methods will work fine. The challenge for the test is to work fine for the other cases as well.

The log-linear simulation model is closely related to Goodman's Row-Column (RC) model (Goodman 1979) and was first used in ecology by Ihm and van Groenewoud (1975); see ter Braak (2014) and Jamil and ter Braak (2013) for its ramifications and links to the Gaussian response model and correspondence analysis.

In the description of the model it is suggested that the random variables *x* and *z* are latent (unobserved) environmental and trait variable, respectively. Another interesting interpretation of the model is that it is a generalized linear mixed model (GLMM) for trait-environment association as formulated by Jamil et al. (2013). They started from a model relating abundance to the environment *e* by a GLM model, which is in the current setting a log-linear model of abundance against environment of the form

$$\log(\mu_{ij}) = \gamma_i + \alpha_j + \beta_j e_i. \qquad \text{(A.4)}$$

In Jamil et al. (2013) the parameter γ_i was taken random as they dealt primarily with binary data, but in the count-case it can also be taken fixed in GLM with a pseudo-Poisson likelihood (Gourieroux et al. 1984a; Gourieroux et al. 1984b). The parameters α_j and β_j are the intercept and the slope of the log-linear relation between abundance and environment. This RC model has both linear and unimodal properties (Jamil & ter Braak 2013). The next step in Jamil et al. (2013) was to investigate whether the intercept and slope, which are both species-specific, are related to a specific trait *t* by the linear regression models

$$\alpha_j = a_0 + a_1 t_j + \epsilon_{\alpha j} \text{ and } \beta_j = b_0^* + b_1^* t_j + \epsilon_{\beta j} \quad \text{(A.5)}$$

with a_0, a_1, b_0^* and b_1^* unknown parameters and $\epsilon_{\alpha j}$ and $\epsilon_{\beta j}$ (possibly correlated) normal random variables with variance σ_α^2 and σ_β^2. There is trait-environment association if b_1^* is nonzero. After insertion of these equations in the previous one, the model becomes

$$\log(\mu_{ij}) = \gamma_i + a_0 + a_1 t_j + \epsilon_{\alpha j} + (b_0^* + b_1^* t_j + \epsilon_{\beta j}) e_i$$

$$= a_0 + (\gamma_i + b_0^* e_i) + (a_1 t_j + \epsilon_{\alpha j}) + b_1^* t_j e_i + \epsilon_{\beta j} e_i$$

$$= \mu_0 + R_i + C_j + b_1 t_j e_i + b_2 z_j e_i \quad \text{(A.6)}$$

with $\mu_0 = a_0, R_i = \gamma_i + b_0^* e_i, C_j = a_1 t_j + \epsilon_{\alpha j}, b_{te} = b_1^*, z_j = \epsilon_{\beta j}/\sigma_\beta$ and $b_{ze} = \sigma_\beta$, which is a special case of the log-linear simulation model with $b_{tx} = 0$ and $\sigma_\epsilon^2 = 0$.

Unless noted explicitly otherwise, we used in our simulations

- $\mu_0 = \log(30)$,
- $R_i = 0.05 e_i - 0.1 e_i^2 + \varepsilon_{ri}$ with ε_{ri} an independent normal random variable: $\varepsilon_{ri} \sim N(0, 0.01)$, giving an common Gaussian response component with optimum at 0.25 and tolerance 2.24,
- $C_j = 0.05 t_j - 0.1 t_j^2 + \varepsilon_{tj}$ with ε_{tj} an independent normal random variable: $\varepsilon_{tj} \sim N(0, 0.01)$, giving an common Gaussian response component with optimum at 0.25 and tolerance 2.24,
- $b_{zx}^* = 0.2$ and $\sigma_\epsilon^2 = 0.2^2$ to give both structured and unstructured interaction among species and sites that is unrelated to the trait-environment association between $\{t_j\}$ and $\{e_i\}$.

References

Dray, S., and P. Legendre. 2008. Testing the species traits environment relationships: The fourth-corner problem revisited. Ecology **89**:3400-3412.

Goodman, L. A. 1979. Simple models for the analysis of association in cross-classifications having ordered categories. Journal of the American Statistical Association **74**:537-552.

Gourieroux, C., A. Monfort, and A. Trognon. 1984a. Pseudo Maximum Likelihood Methods: Applications to Poisson Models. Econometrica **52**:701-720.

Gourieroux, C., A. Monfort, and A. Trognon. 1984b. Pseudo Maximum Likelihood Methods: Theory. Econometrica **52**:681-700.

Ihm, P., and H. v. Groenewoud. 1975. A Multivariate Ordering of Vegetation Data Based on Gaussian Type Gradient Response Curves. Journal of Ecology **63**:767-777.

Jamil, T., W. A. Ozinga, M. Kleyer, and C. J. F. ter Braak. 2013. Selecting traits that explain species–environment relationships: a generalized linear mixed model approach. Journal of Vegetation Science **24**:988-1000.

Jamil, T., and C. J. F. ter Braak. 2013. Generalized linear mixed models can detect unimodal species-environment relationships. PeerJ **1**:e95.

Peres-Neto, P.R., S. Dray, and C. ter Braak. 2016. Linking trait variation to the environment: critical issues with community-weighted mean correlation resolved by the fourth-corner approach. Ecography. http://dx.doi.org/10.1111/ecog.02302.

Swan, J. M. A. 1970. An Examination of Some Ordination Problems By Use of Simulated Vegetational Data. Ecology **51**:89-102.

ter Braak, C. J. F. 2014. History of canonical correspondence analysis. Pages 61-75 *in* J. Blasius and M. Greenacre, editors. Visualization and verbalization of Data. Chapman and Hall/CRC, London.

ter Braak, C. J. F., A. Cormont, and S. Dray. 2012. Improved testing of species traits–environment relationships in the fourth-corner problem. Ecology **93**:1525-1526.
