# Supplementary material for: A critical issue in model-based inference for studying trait-based community assembly and a solution
Source: PeerJ. 2017 Jan 12;5:e2885. doi: 10.7717/peerj.2885 (PMC5237366; doi:10.7717/peerj.2885)
Supplement: Appendix S2 — Description of the R functions and script with example output. [file peerj-05-2885-s002.pdf]

A critical issue in model-based inference for studying trait-based community assembly and a solution

Cajo J. F. Ter Braak, Pedro R. Peres-Neto and Stéphane Dray

Appendix S2

2. R functions and example script

This appendix gives R functions, code and output log supporting the main conclusions of the paper.

2.1 Data generating functions

The key functions are:

generate_Tdata – generates multivariate normal trait data and a linear combination thereof that is standard normally distributed.

generate_community_Gaussian_response – implements the Gaussian response model of Appendix S1.1 with a response distribution that defaults to negative-binomial ("negbin"). The other allowed distributions are: "poisson" (which allows zero-inflation by the argument nu), "binomial" for presence/absence data, and "ZINBI", for the zero-inflated negative-binomial with argument nu for the fraction of negative-binomial draws set afterwards to zeros. The randomness in the species-specific tolerance (σ_j, sigma in the R code) make that the model is not a reparametrized submodel of the next log-linear model.

generate_RC_community – implements the log-linear model of Appendix S1.2 with for completeness also the term + $b_{zx} z_j x_i$ in the linear predictor. In the example script b_{zx} = 0.

Save the following code as R-file with name: generate_trait_env_community_data.r

```
# function to generate one- and multi-trait data ----------------------------------
-----
generate_Tdata <-function(n_species,q, correlation = 0, b){
  # n_species = number of species; q = number of traits;
  # correlation = AR(1) autocorrelation between subsequent variables
  # b = coefficient of the linear combination (c in the paper)
  # generate q correlated normally distibuted trait variables in Tdat
  # a linear combination of these  Tdat%*%b  in T
  # rescale b and T so that T ~ N(0,1),
  # value: list with Tdat, T and coefficients (rescaled b)
  ii <- 1:q
  Sigma <-  correlation^abs(outer(ii,ii,  '-'))
  sdE <-  sqrt(b %*% Sigma %*%b)
  coefs <- b/sdE
  Tdat<-MASS::mvrnorm(n_species,mu=rep(0,q),Sigma)
  T <- Tdat %*%coefs
  rownames(T)<- rownames(Tdat)<- paste("spec", seq_len(nrow(Tdat)), sep="")
  return(list(T = T, Tdat= Tdat, coefficients = coefs))
}
```

```
# function to generate (zero inflated ) negative binomial data ------------
ZInbinom <- function(mu,sigma = 1, nu= 0){
  L <- rnbinom(length(mu), size = 1/sigma, mu = mu)
  # variance = mu + mu^2/size = mu + sigma * mu^2
  L <- ifelse(runif(length(mu)) < nu,  0,L)
  matrix(L,nrow = nrow(mu),ncol = ncol(mu))
}

# function to generate (zero inflated ) poisson data ------------
ZIpoisson <- function(mu, nu= 0){
  L <- rpois(length(mu), lambda = mu)
  L <- ifelse(runif(length(mu)) < nu,  0,L)
  matrix(L,nrow = nrow(mu),ncol = ncol(mu))
}



# function to generate data according to the Gaussian response model Eqn A1.1 and y
~NegBin-----------------------------------------------
generate_community_Gaussian_response <- function(tolerance=2,E,T,n_species = length
(T),n_communities = length(E), mu_max = 10,  distribution = "negbin", sigma = 1, nu
= 0, fct = 0.8, max.try = 5){
  # using outer instead of a for loop
  # tolerance sets the maximum width of the random sigma_j in the paper (set to 2 i
n the paper)
  # with checks on empty rows and columns
  # the repeat loop is to ensure that the final number of  species and sites is wit
hin fct (0.8) of the number asked for in  argument
    i.try <- 0
  repeat {
    i.try <- i.try + 1
    # one trait, one environmental variable
    E <- c(E)
    T <- c(T)
    h<-runif(n_species,min=0.3,max=1)
    sigma<-runif(n_species)*tolerance
    mu <- outer(rep(mu_max,n_communities),h) * exp(outer(c(E),c(T),FUN = "-")^2 / o
uter(rep(1,n_communities),-2*sigma^2))+1.e-10
    L <- switch( distribution,
                 poisson = ZIpoisson(mu,nu=nu),
                 binomial = matrix(rbinom(prod(dim(mu)),1, mu),nrow = nrow(mu),ncol
= ncol(mu)),
                 negbin  = ZInbinom(mu = mu, sigma= sigma, nu = 0),
                 ZINBI =  ZInbinom(mu = mu, sigma=sigma, nu =nu)
    )
    n_species_c<-sum(colSums(L)!=0) # _c for check
    n_communities_c<-sum(rowSums(L)!=0)
    if ((n_species_c >= fct * n_species) && (n_communities_c >=fct *n_communities |
| i.try > max.try)){break}
  }
  rownames(L)<- paste("site", seq_len(nrow(L)), sep="")
  colnames(L)<- paste("spec",seq_len(ncol(L)),sep="")
  return(L)  # L = Y in the paper
}


# function to generate data: RC-model/ loglinear model with y~NegBin eqn A.3-------
-------------
generate_RC_community <- function(E,T, coefs=c(0,0.2,0,0,0),
                                  sigma_epsilon_ij = 0.2, mu0 =30,
                                  coefsE=c(0.05, -0.1, 0.1),  coefsx=c(0,0,0),
                                  coefsT=c(0.05, -0.1, 0.1),  coefsz=c(0,0,0),
                                  distribution = "negbin", link.mu = "log",
                                  sigma = 1, nu = 0,
```

```
                                fct = 0.8, max.try = 5){
  #
  # one environmental variable E, one trait T
  # main effects:
  #    row and column effects functions
  #       f_row and f_col: a polynomial in E, T,x and z respectively
  #           d1*E + d2*E^2 + d3*rnorm(n, 0,1), with coefsE = c(d1,d2,d3)
  #           d1*T + d2*T^2 + d3*rnorm(n, 0,1), with coefsT = c(d1,d2,d3)
  #           d1*x + d2*x^2 + d3*rnorm(n, 0,1), with coefsx = c(d1,d2,d3)
  #           d1*z + d2*z^2 + d3*rnorm(n, 0,1), with coefsz = c(d1,d2,d3)
  #           added noise if d3 is nonzero
  # interaction effects
  #        b1*T*E + b2*z*E + b3*T*x + b4*x*z+ b5*xx*zz eps with coefs = c(b1,b2,b3,b4)
  #        with z ~ N(0,1), x~N(0,1), zz ~ N(0,1), xx~N(0,1)and eps~sigma_epsilon_ij*N(0,1)
  #        Note:  trait-environment association only if b1 !=0
  #        Note:  confounding random effects if coefs[2:3] are non-zero
  #
  # dist ="negbin", "ZINBI" , "pois", "binomial"
  # link =  link function "log", "logit"
  # if center. = TRUE then the interaction is double centered
  # the repeat loop is to ensure that the final number of  species and sites is within fct (0.8) of the number asked for in  argument

  # The equi-tolerance versions of the Gaussian models
  # of Peres-Neto, Dray & ter Braak (2016, Ecography http://dx.doi.org/10.1111/ecog.02302) and
  # ter Braak, Peres-Neres & Dray (2016, PeerJ) can be simulated
  # by setting coefsE-coefsz and coef as follows:
  # The environmentally structured, random trait case corresponds to
  # coefsE=c(0,-0.5/tolerance^2, 0);  coefsx=c(0,0,0)
  # coefsT=c(0,0, 0);  coefsz=c(0,-0.5/tolerance^2,0)
  # The trait structured, random environment case corresponds to
  # coefsE=c(0,0, 0);  coefsx=c(0,-0.5/tolerance^2,0)
  # coefsT=c(0,-0.5/tolerance^2, 0);  coefsz=c(0,0,0)
  # The both random case corresponds to
  # coefsE=c(0,0, 0);  coefsx=c(0,-0.5/tolerance^2,0)
  # coefsT=c(0,0, 0);  coefsz=c(0,-0.5/tolerance^2,0)

  #  local functions: marginal effects
  f_row <- function(E, coefsE){
    effects <- coefsE[1]*E + coefsE[2]*E*E + coefsE[3]*rnorm(length(E))
    effects
  }
  f_col <- function(T, coefsT){f_row(T,coefsE=coefsT)}
  # end local functions
  E <- c(E);
  T <- c(T)
  n_communities <- length(E)
  n_species <- length(T)
  i.try <- 1
  if (length(coefs)==3) coefs <- c(coefs,0,0)
  if (length(coefs)==4) coefs <- c(coefs,0)
  repeat {
    i.try <- i.try + 1
    x <-  rnorm(length(E)); z <-  rnorm(length(T))
    xx <-  rnorm(length(E)); zz <-  rnorm(length(T))
    mu.interaction <-
      coefs[1] * outer(E,T,FUN = "*") +
      coefs[2] * outer(E,z,FUN = "*") +
      coefs[3] * outer(x,T,FUN = "*") +
      coefs[4] * outer(x,z,FUN = "*") +
      coefs[5] * outer(xx,zz,FUN = "*") +
      sigma_epsilon_ij * matrix(rnorm(n_communities*n_species), nrow =n_communities)
```

```
    mu.marginal <- outer(f_row(E,coefsE), f_col(T,coefsT), FUN="+")
    mu.lp <- mu.marginal + mu.interaction
    # for numerical stability we needed to limit mu.lp
    mu.lp <- ifelse(mu.lp > 10, matrix(10, nrow=nrow(mu.lp),ncol=ncol(mu.lp)), mu.l
p)
    mu.lp <- ifelse(mu.lp < -10,matrix(-10, nrow=nrow(mu.lp),ncol=ncol(mu.lp)), mu.
lp)
    if (mu0 > 0.9 && (distribution =="binomial"|| link.mu =="logit"))  mu0 <- 0.5 #
    mu <- switch(link.mu,
                 log =  mu0 * exp(mu.lp),
                 logit = 1/(1 + exp(-( mu.lp + log(mu0/(1-m0)))) )
    )
    L <- switch( distribution,
                 poisson = ZIpoisson(mu,nu=nu),
                 binomial = matrix(rbinom(prod(dim(mu)),1, mu),nrow = nrow(mu),ncol
= ncol(mu)),
                 negbin  = ZInbinom(mu = mu, sigma= sigma, nu = 0),
                 ZINBI =  ZInbinom(mu = mu, sigma=sigma, nu =nu)
    )
    nspecies_c <- sum(colSums(L)!=0) # _c for check
    ncommunities_c <- sum(rowSums(L)!=0)
    if ((nspecies_c >= fct * n_species) && (ncommunities_c >=fct *n_communities ||
i.try> max.try)){break}
  }
  rownames(L)<- paste("site", seq_len(nrow(L)), sep="")
  colnames(L)<- paste("spec",seq_len(ncol(L)),sep="")
  return(L) # L = Y in the paper
}
```

2.2 R functions for trait-environment testing

The key functions are:

make_obj_for_traitenv - makes object of class “TE_obj” from matrices or dataframes from L,E1 and T1; no factors allowed, as all is converted to matrices; the object is a list of three matrices: L, E, T where L takes the role of Y in the paper.

anova_traitglm – use library `mvabund` for doing anova.traitglm with calling of traitglm. The function uses try() to try and catch error, which sometimes helps avoiding failures in simulations.

pmax_PermutationTest – implements the $p_{\max}$ test for an arbitrary test statistic using the argument `FUN_test_statistics`. The functions for generating such test statistics are the next two functions. The first is the default.

trait_env_glm_LR – calculates the likelihood ratio (in fact the deviance) of the null model with only site and species main effects (row and column effects) and the alternative model with all interactions between E and T. The function can deal with one environmental variable and one to many quantitative traits. With more than one trait, this amount to a joint test on the interactions of e with the traits.

Fourthcorner - calculates the squared fourth-corner correlation for quantitative traits and environmental variables. For more than one trait and one environmental variable, it gives the sum of their squared fourth-corner correlations. This is the total inertia in an RLQ analysis (Dray et al. 2014; Jamil et al. 2013).

Helper functions are:

df2matrix – turns a list of data frames into matrices

matrix2df – turns a list of matrices into data frames

expand4glm – vectorizes the abundance data table L (Y) to one long vector and generates associated factor of sites and species and the associated trait and environmental variables (adapted from (Jamil et al. 2013)).

#fourthcornerglmm0 – as anova_traitglm, but now for the GLMM (adapted from (Jamil et al. 2013)). The function is commented out not used in the example script.

test_statistics_TE – generates two test statistics for uses with `pmax_PermutationTest`. It combines `trait_env_glm_LR` and `Fourthcorner`.

Save the following code as R-file with name: te_test_functions.r

```
# make object from data (L=Y) with checks on empty row/cols -----------------------
----------

make_obj_for_traitenv <- function(L,E1,T1, cut_off = 0){
  # makes TE_obj_matrices from L,E1 and T1
  # the object is a list of three matrices: L, E, T
  L <- as.matrix(L)
  E1 <- as.matrix(E1)
  T1 <- as.matrix(T1)
  # check_L()
  rows<-seq_len(nrow(L))
  cols<-seq_len(ncol(L))
  rni <-which(rowSums(L)==0)
  repeat {
    if (length(rni)) {L <- L[-rni,,drop = FALSE]; rows <-rows[-rni]}
    ksi <- which(colSums(L)==0)
    if (length(ksi)) {L <- L[,-ksi, drop = FALSE]; cols <- cols[-ksi]}
    rni <-which(rowSums(L)==0)
    if ( length(rni)==0 & length(ksi)==0){break}
  }
  E1 <-as.matrix(E1)[rows,,drop = FALSE]
  T1 <-as.matrix(T1)[cols,,drop = FALSE]
  # end check_L()
  if(cut_off >0 ){
    abOcc <- apply(L>0,2,mean)
    L <- L[,abOcc>cut_off, drop =FALSE]
    T1 <- as.matrix(T1)[abOcc>cut_off,, drop = FALSE]
  }
  if(is.null(rownames(L)))rownames(L)<- paste("site", 1:nrow(L), sep="")
  if(is.null(colnames(L)))colnames(L)<- paste("spec",1:ncol(L),sep="")
  rownames(E1)= rownames(L); colnames(E1)=  paste("E", 1:ncol(E1), sep = "")
  rownames(T1)= colnames(L); colnames(T1)=  paste("T", 1:ncol(T1), sep = "")
  obj = list(L=L, E=E1,T = T1)
  class(obj) = c("TE_obj", class(obj))
  return(obj)
}


# convert lists of data frames to matrices and the reverse ----------------
```

```
df2matrix <- function(obj){
  # makes matrices from the data frames in obj
  lapply(obj,as.matrix)
}

matrix2df <- function(obj){
  # makes dataframes from the matrices in obj
   lapply(obj,as.data.frame)
}


# function doing traitglm and anova.traitglm on obj -----------------------

anova_traitglm <- function(obj, family = "negative.binomial", composition=TRUE, nre
pet = 39){
  # obj is from make_obj_for_traitenv or its dataframe version
  # nrepet is number of bootstraps in anova.traitglm
  # value:  c("Devdiff","pval")
  if (class(obj)[1]=="TE_obj") obj <- matrix2df(obj)
  with(obj,{
    ft0 <- try(traitglm(L,E,T,formula = ~1, family= family, method="manyglm", compo
sition=composition))
    # ft1 <- try(traitglm(L,E,T,formula = ~E1:T1, family= family, method="manyglm",
composition=composition))
    # use all interactions: no formula!
    # we need  try() here as sometimes there is trouble. Even this does not work aw
ays.
    ft1 <- try(traitglm(L,E,T, family= family, method="manyglm", composition=compos
ition))
    if (class(ft0)[1]=="traitglm" && class(ft1)[1]=="traitglm"){
      Devdiff <- ft1$two.loglike - ft0$two.loglike #== Dev in anova.manyglm(ft0,ft1
,nBoot=1)
      an <- try(anova.traitglm(ft0,ft1,nBoot=nrepet, show.time = "none")  )
      if (class(an)=="anova.manyglm") pval  <- an$table$"Pr(>Dev)"[2] else pval <-
NA
      result <- c(Devdiff, pval)
    } else result <- c(NA,NA)
    names(result)= c("Devdiff","pval")
    return(result)
  })
}

# special purpose functions for glm replacing traitglm ----------------------------
------

# 1. expand the data in the obj (L,E,T) in vector form -----------------------
expand4glm <- function(obj){
  if (class(obj)[1]=="TE_obj") obj <- matrix2df(obj)
  # adapted from Jamil et al 2013; one environmental variable only, 1-many traits!
  with(obj, {
    sitespec <- expand.grid(rownames(L),colnames(L))
    site <-sitespec[,1]; species<-sitespec[,2]
    y <- as.vector(as.matrix(L))
    Evec <-  E[site,, drop = FALSE];  rownames(Evec)= NULL
    Tvec <-  T[species,, drop = FALSE]; rownames(Tvec)= NULL
    # for one Evec!!!
    ET <- Tvec * Evec[,1]; colnames(ET)= paste("E", colnames(T),sep="")
    XYZ <- data.frame(y,site,species,ET)
    return(XYZ) # XYZ notation of Jamil et al 2013
  })
}

trait_env_glm_LR <- function(obj, family_ = poisson, composition=TRUE, fitH0){
  # trait_env_glm_LR calculates the deviance difference (=2log(LR)) between
  # the null (R+C) model and the R+C+et model for resampling
  # the R+C null model does not depend on E and T and thus remains constant during
```

```
resampling
  # used via fitH0
  #
  # obj: objectfrom make_obj_for_traitenv for all preprocessing
  # fitH0: list(deviance, eta, formula_with_ET) with deviance and eta (linear predi
ctor) of null model
  #           and the formula for the alternative model (fitH0$formula_with_ET)
  # value: the deviance differene as used in anova.manyglm
  XYZ <- expand4glm(obj)
  fit_with_ET <- glm(fitH0$formula_with_ET, family = family_, data = XYZ, etastart
= fitH0$eta)
  Devdiff <-  fitH0$deviance - fit_with_ET$deviance
  return(c(glmRCmax=Devdiff))
}

pmax_PermutationTest <- function(obj, FUN_test_statistics=trait_env_glm_LR, nrepet
= 39, withglm=TRUE, ...) {
  # obj from make_obj_for_traitenv(L,E,T, cut_off): matrices L,E and T (one environ
mental variable, 1-many traits)
  # FUN_test_statistic : function that returns one or more test statistics
  # e.g.FUN_test_statistic = trait_env__glm_LR or test_statistics_TE;
  # ... options for FUN_test_statistic
  # NOTE: the reuse of the null model (fitH0 below) is only valid with compositiona
l is true, or without any row main effects/ any column main effects
  # function uses: composition == TRUE

  if  (withglm){
    XYZ <- expand4glm(obj) # data frame XYZ
    #names(XYZ)
    gl0 <- glm(y~site+species, family = poisson, data = XYZ)
    formula_with_ET <- as.formula(paste( "y~site+species+",paste(paste("ET", 1 : nc
ol(obj$T),sep =""),collapse = "+"),sep=""))
    fitH0 <- list(deviance= gl0$deviance, eta = gl0$linear.predictors, formula_with
_ET=formula_with_ET)
  } else fitH0 = NA
  obs <- FUN_test_statistics(obj,fitH0=fitH0, ...)
  sim.row <- matrix(0, nrow = nrepet, ncol = length(obs))
  sim.col <- matrix(0, nrow = nrepet, ncol = length(obs))
  #sim.rc <- matrix(0, nrow = nrepet, ncol = length(obs))
  for(i in 1:nrepet){
    per.row <- sample(nrow(obj$L))
    per.col <- sample(ncol(obj$L))
    #permute_rows_columns <- function(obj, per.row,per.col){
    obj.row <- obj.col <-   obj   # obj.rc <- obj
    obj.row$E <-obj.row$E[per.row,,drop =FALSE]
    obj.col$T <-obj.col$T[per.col,,drop =FALSE]
    sim.row[i, ] <- FUN_test_statistics(obj.row,fitH0=fitH0, ...)
    sim.col[i, ] <- FUN_test_statistics(obj.col,fitH0=fitH0, ...)
  }
  if (length(obs)==1){
    pval.row <- (sum(abs(sim.row) >= abs(obs), na.rm = TRUE) + 1)  / (nrepet + 1)
    pval.col <- (sum(abs(sim.col) >= abs(obs), na.rm = TRUE) + 1)  / (nrepet + 1)

  } else {
    pval.row <- (rowSums(apply(abs(sim.row), 1, function(i) i >= abs(obs)), na.rm =
TRUE) + 1)  / (nrepet + 1)
    pval.col <- (rowSums(apply(abs(sim.col), 1, function(i) i >= abs(obs)), na.rm =
TRUE) + 1)  / (nrepet + 1)
  }
  result <- cbind(test_stat = obs, prow = pval.row, pcol = pval.col, pmax = pmax(pv
al.row, pval.col))
  return(result)
}
```

```
# # function using parametric glmm (similar to anova_traitglm, but no sampling)----
------------
# library(lme4)
# fourthcornerglmm0 <- function(obj, family.glmm=poisson(link = "log")){
# # determine the parametric test on interaction from the glmm of Jamil et al 2013
for count data
# #  obj <- make_obj_for_traitenv(L,E,T, cut_off)
# #  obj <- expand4glmm(obj)
#   if (is.matrix(obj$L)) obj <- matrix2df(obj)
#   if (length(obj)==3) obj <- expand4glmm(obj)
#   fm0 = try(glmer(y ~ site + species + (0 + E1 | species)+ (1|site.spec),
#               family=family.glmm,data=obj))
#   fm1 = try(update(fm0, formula= . ~. + E1:T1 ))
#   if( class(fm0)=="glmerMod" && class(fm1)=="glmerMod" ){
#     an <- anova(fm1,fm0)
#     pval <- an$"Pr(>Chisq)"[2]
#     Devdiff <-an$deviance[1]- an$deviance[2]
#   } else { pval <- NA; Devdiff <- NA}
#   result <- c(Devdiff=Devdiff, pval=pval)
#   names(result)= c("Devdiff","pval")
#   return(result)
# }

# fourth corner test statistic (could use ade4 package instead... )----------------
----------------------------
fourthcorner <- function(obj,...){
  # fourthcorner function for resampling for numeric data
  # obj is from make_obj_for_traitenv for all preprocessing
  # ... added to allow fourthcorner as FUN_test_statistics in pmax_PermutationTest
  # value: the sum of the squares of the fourthcorner correlation(s) between E and
T (can be matrices)
  #
  # local functions
  center_w <- function(X,w = rep(1/nrow(X),nrow(X))){ X - rep(1,length(w))%*%t(w)%*
% X }
  standardize_w <- function(X,w = rep(1/nrow(X),nrow(X))){
    ones <- rep(1,length(w))
    Xc <- X - ones %*% t(w)%*% X
    Xc / ones%*%sqrt(t(ones)%*%(Xc*Xc*w))
  }
  # end of local functions
  #if(is.data.frame(obj$L)) obj <- df2matrix(obj)
  with(obj,{
    L<-L/sum(L)
    Wn <- rowSums(L)
    Ws <- colSums(L)
    Estd_w <- standardize_w(E,Wn)
    Tstd_w <-  standardize_w(T,Ws)
    # Fourth corner calculated as W_n weighted covariance between
    Fourthcorner <- t(Estd_w)%*%(L%*%Tstd_w)
    Fourthcorner <- sum(Fourthcorner*Fourthcorner)
    return(Fourthcorner=Fourthcorner)
  })
}


test_statistics_TE <- function(obj, family=poisson,  fitH0){
  # function to generate test statistics from an obj from make_obj_for_traitenv (TE
_obj object)
  # for use in RC_pmax_PermutationTest
  # value: a named vector of all test statistics
  fourth <- fourthcorner(obj)
  trglm <- trait_env_glm_LR(obj, family, composition=TRUE, fitH0=fitH0)
  res <- c(trglm, fourth)
  names(res)= c("glm","fourth")
```

```
  return(res)
}
```

2.3 Example R script

The script contains the sections, all simulating null models:

Section 1a Gaussian response simulations with 10 random traits

Section 1b Gaussian response simulations with one random trait

Section 2a log-linear simulation model b_tx = 0

Section 2b log-linear simulation model b_tx != 0 single trait

Section 2c log-linear simulation model b_tx != 0 10 random traits

The first three sections are 'trait random' cases and the last two are 'both random interaction' cases.

The methods studied are

(1) `anova.traitglm`, using the function `anova_traitglm` that class traitglm and anova.traitglm, the $p_{\max}$ test using (2) glm and (3) fourth-corner with the function `pmax_PermutationTest` that uses `trait_env_glm_LR` and `fourthcorner` for calculating the Poisson-based likelihood ratio (LR) and the (sum of) squared fourth-correlation(s), respectively. In the script, the number of sites is set to 20, the number of species to 11 and the number of simulations to 10 so as to minimize computing time for showing the main results of the paper.

The purpose of Sections 1a (10 random traits) is to show that `anova.traitglm` can give very high type I error. In the remaining sections the bad behavior of `anova.traitglm` is taken for granted and the focus on the type I error control of the proposed solution, the $p_{\max}$ test. Here the number of simulations is 1000. With the lower number of species and sites, the type I error is only slightly inflated (type I error of 0.06) in section 2b and, with 10 random traits, no inflation.

```
rm(list=ls(all=TRUE))  # remove all existing items from the workspace
library(mvabund)
source("generate_trait_env_community_data.r")
source("te_test_functions.r")


# Section 1a Gaussian response simulations with 10 random traits ------------------
--------------------------------
# n = 20; m = 11; q = 10
n_communities <- 20 #n
n_species <- 11 # m
mu_max <- 10
n_traits <- 10  #q
cor_Tdat <- 0


n_simul <-  10  # in the paper we did 1000 unless otherwise noted.
nrepet <- 39
```

```
nominal_level <-  0.05
(threshold <- (nominal_level + 2*sqrt(nominal_level*(1-nominal_level)/n_simul)))

# Gaussian response model settings
mu_max <- 10; tolerance = 2
rho_T = 0; rho_E = 1 # Eqn 1 and Eqn A.1/A.2

set.seed(1257)

pvals <- matrix(NA, nrow = n_simul, ncol = 7);
colnames(pvals)=c("anova.traitglm", "glm_prow", "glm_pcol", "glm_pmax","4th_prow",
"4th_pcol", "4th_pmax")

for (i in 1:n_simul){
    Tlist <- generate_Tdata(n_species, n_traits, cor_Tdat, b = rep(1,n_traits))
    Tdat <- Tlist$Tdat; T <-Tlist$T; c.true <-Tlist$coefficients
    E <- as.matrix(rnorm(n_communities))
    z <- rnorm(n_species); x <- rnorm(n_communities)
    z_star <- rho_T * T + sqrt(1 - rho_T^2)*z # rho_T = 0 == trait random; Eqn A.2
    x_star <- rho_E * E + sqrt(1 - rho_E^2)*x # rho_E = 1 == env NOT random  Eqn A.
2
    # thus: as rho_T = 0 and rho_E = 1
    # z_star = z # the trait governing the Gaussian model, but, alas, unobserved (l
atent)
    # x_star = E # the observed trait that is also the one governing the Gaussian m
odel
    Y <- generate_community_Gaussian_response(tolerance = tolerance, E = x_star, T
= z_star, mu_max = mu_max)
    obj <- make_obj_for_traitenv(Y,E,Tdat)
    res_anova.traitglm <- anova_traitglm(obj, nrepet = nrepet)
    res_glm_pmax <- pmax_PermutationTest(obj, trait_env_glm_LR, nrepet = nrepet)
    res_4th_pmax <- pmax_PermutationTest(obj, fourthcorner, nrepet = nrepet, withgl
m = FALSE)
    pvals[i,]= c(res_anova.traitglm["pval"], res_glm_pmax[-1],res_4th_pmax[-1])
}
#warnings() # glm.fit: fitted rates numerically 0 occurred
rejection_rate_q = colMeans(1*(pvals  <= nominal_level))
suspect.rej<- rejection_rate_q  > threshold
print(rejection_rate_q)
# # anova.traitglm       glm_prow       glm_pcol       glm_pmax       4th_prow
# # 0.9            0.7            0.0            0.0            0.6
# # 4th_pcol       4th_pmax
# # 0.1            0.1

print(suspect.rej)
# # anova.traitglm       glm_prow       glm_pcol       glm_pmax       4th_prow
# # TRUE            TRUE           FALSE          FALSE           TRUE
# # 4th_pcol       4th_pmax
# # FALSE          FALSE
# # Conclusion 1: anova.traitglm gives highly elevated type I error (detectable eve
n with n_simul = 10!)
# # Conclusion 2: glm_pmax gives controls the type I error rate
# # Conclusion 3: 4th_pmax gives controls the type I error rate
# # Remark: Inspection of pvals shows that in this many traits case,
# # glm and 4th (actually the RLQ intertia) differ on a per data set basis;
# # this is not only due different in the permutation, but is generally expected,
# # particularly so when cor_Tdat!=0 or the linear combination b does not simply sp
ecify a sum

# Section 1b Gaussian response simulations with one random trait ------------------
--------------------------------

pvals <- matrix(NA, nrow = n_simul, ncol = 7);
colnames(pvals)=c("anova.traitglm", "glm_prow", "glm_pcol", "glm_pmax","4th_prow",
"4th_pcol", "4th_pmax")
```

```
for (i in 1:n_simul){
  T <- as.matrix(rnorm(n_species))
  E <- as.matrix(rnorm(n_communities))
  z <- rnorm(n_species); # x <- rnorm(n_communities)
  z_star <- rho_T * T + sqrt(1 - rho_T^2)*z # rho_T = 0 == trait random; Eqn A.2
  x_star <- rho_E * E + sqrt(1 - rho_E^2)*x # rho_E = 1 == env NOT random  Eqn A.2
  # thus: if rho_T = 0 and rho_E = 1
  # z_star = z # the trait governing the Gaussian model, but, alas, unobserved (lat
ent)
  # x_star = E # the observed trait that is also the one governing the Gaussian mod
el
  Y <- generate_community_Gaussian_response(tolerance = tolerance, E = x_star, T =
z_star, mu_max = mu_max)
  obj <- make_obj_for_traitenv(Y,E,T)
  res_anova.traitglm <- anova_traitglm(obj, nrepet = nrepet)
# # in the next three lines, independent row and columns permutations are used for
the two tests
#   res_glm_pmax <- pmax_PermutationTest(obj, trait_env_glm_LR, nrepet = nrepet)
#   res_4th_pmax <- pmax_PermutationTest(obj, fourthcorner, nrepet = nrepet)
#   pvals[i,]= c(res_anova.traitglm["pval"], res_glm_pmax[-1],res_4th_pmax[-1])
# # in the next line below, the same row and columns permutations are used for the
two tests
# # so as to show that they give about the same result if n_env=n_traits=1
  glm_4th <- pmax_PermutationTest(obj, test_statistics_TE, nrepet = nrepet)
  pvals[i,]=c(res_anova.traitglm["pval"],glm_4th["glm",-1],glm_4th["fourth",-1])
}
#warnings() # glm.fit: fitted rates numerically 0 occurred
rejection_rate_1 = colMeans(1*(pvals  <= nominal_level))
suspect.rej<- rejection_rate_1  > threshold
print(rejection_rate_1)
# anova.traitglm       glm_prow       glm_pcol       glm_pmax       4th_prow
# 0.3            0.3            0.0            0.0            0.3
# 4th_pcol       4th_pmax
# 0.0            0.0
print(suspect.rej)
# # anova.traitglm       glm_prow       glm_pcol       glm_pmax       4th_prow
# # TRUE            TRUE           FALSE          FALSE           TRUE
# # 4th_pcol       4th_pmax
# # FALSE          FALSE
# # Conclusion 1: anova.traitglm gives elevated type I error (detectable even with
n_simul = 10!)
# # Conclusion 2: glm_pmax gives controls the type I error rate
# # Conclusion 3: 4th_pmax gives controls the type I error rate
# # Remark: Inspection of pvals shows that in this one-one case,
# # glm and 4th do hardly differ on a per data set basis
pvals[, 1+(1:3)]- pvals[,4+(1:3)]
# # summary:
id = apply(abs(pvals[, 1+(1:3)]- pvals[,4+(1:3)]) > 0, 1, sum)
sum(id);  # unequal in one data set
which(id >0) # namely the seven-th data set, if which(id >0) = 7
pvals[id,]

rm(z, z_star, x_star, Y, E, T)

# Section 2a log-linear simulation model b_tx = 0 ---------------------------------
------------

mu0 = 30
# effect sizes in log-linear model (b1,b2,b2 = b_te, b_ze, b_tx)
# with x and z random nuisance variables.
b_te <- 0   # the null model, without t-e interaction
b_ze <- 0.8
b_tx <- 0
b_zx <- 0
b_zx_star <- 0.2
sigma_epsilon_ij <- 0.2 # min(0.1, b_te + b_ze + b_tx)   # unstructured interaction
```

```r
ef.main <- 0.05  # runif(1,min=0.1, max = ef.main)
ef.main.sq <-  -0.1
ef.main.ran.rows <- 0.1
ef.main.ran.columns <- 0.1

if (ef.main.sq < 0){
  opt <-  -ef.main/(2*ef.main.sq)
  tol <- 1/sqrt(-2*ef.main.sq)
}
print(opt); print(tol)

pvals <- matrix(NA, nrow = n_simul, ncol = 7);
colnames(pvals)=c("anova.traitglm", "glm_prow", "glm_pcol", "glm_pmax","4th_prow",
"4th_pcol", "4th_pmax")

for (i in 1:n_simul){
  T <- as.matrix(rnorm(n_species))
  E <- as.matrix(rnorm(n_communities))
  Y <- generate_RC_community(E= E, T= T, coefs=c(b_te, b_ze, b_tx, b_zx, b_zx_star)
, sigma_epsilon_ij  = sigma_epsilon_ij, mu0=mu0,
                                 coefsE=c(ef.main,ef.main.sq, ef.main.ran.rows),
                                 coefsT=c(ef.main,ef.main.sq, ef.main.ran.columns))
  obj <- make_obj_for_traitenv(Y,E,T)
  res_anova.traitglm <- anova_traitglm(obj, nrepet = nrepet)
  glm_4th <- pmax_PermutationTest(obj, test_statistics_TE, nrepet = nrepet)
  pvals[i,]=c(res_anova.traitglm["pval"],glm_4th["glm",-1],glm_4th["fourth",-1])
}
#warnings() # glm.fit: fitted rates numerically 0 occurred
rejection_rate_llm = colMeans(1*(pvals  <= nominal_level)) #llm = log-linear model
suspect.rej<- rejection_rate_llm  > threshold
print(rejection_rate_llm)
# anova.traitglm       glm_prow       glm_pcol       glm_pmax       4th_prow
# 0.3            0.2            0.0            0.0            0.2
# 4th_pcol       4th_pmax
# 0.0            0.0
print(suspect.rej)
# anova.traitglm       glm_prow       glm_pcol       glm_pmax       4th_prow
#       TRUE           TRUE          FALSE          FALSE           TRUE
# 4th_pcol       4th_pmax
# FALSE          FALSE
# Conclusion 1: anova.traitglm gives elevated type I error (detectable even with n_
simul = 10!)
# Conclusion 2: glm_pmax gives controls the type I error rate
# Conclusion 3: 4th_pmax gives controls the type I error rate
# Remark: Inspection of pvals shows that in this one-one case,
# glm and 4th do differ slightly on a per data set basis
pvals[, 1+(1:3)]- pvals[,4+(1:3)]
# # summary:
id = apply(abs(pvals[, 1+(1:3)]- pvals[,4+(1:3)]) > 0, 1, sum)
sum(id);  # unequal in ... data sets
which(id >0) # namely the seven-th data set, if which(id >0) = 7
#pvals[id,]


# Section 2b log-linear simulation model b_tx != 0 single trait--------------------
-------------------------

n_simul <- 1000
# simulations with anova.traitglm as it was already shown to be wrong in this kind
of model
# and it takes far too long with 1000 simulations...
mu0 <- 30
# effect sizes in log-linear model (b1,b2,b2 = b_te, b_ze, b_tx)
# with x and z random nuisance variables.
```

```
b_te <- 0   # the null model, without t-e interaction
b_ze <- 0.6
b_tx <- 0.6
b_zx <- 0
b_zx_star <- 0.2
sigma_epsilon_ij <- 0.2 # min(0.1, b_te + b_ze + b_tx)   # unstructured interaction


ef.main <- 0.05  # runif(1,min=0.1, max = ef.main)
ef.main.sq <-  -0.1
ef.main.ran.rows <- 0.1
ef.main.ran.columns <- 0.1

if (ef.main.sq < 0){
  opt <-  -ef.main/(2*ef.main.sq)
  tol <- 1/sqrt(-2*ef.main.sq)
}
print(opt); print(tol)

pvals <- matrix(NA, nrow = n_simul, ncol = 6);
colnames(pvals)=c( "glm_prow", "glm_pcol", "glm_pmax","4th_prow", "4th_pcol", "4th_
pmax")

for (i in 1:n_simul){
  T <- as.matrix(rnorm(n_species))
  E <- as.matrix(rnorm(n_communities))
  Y <- generate_RC_community(E= E, T= T, coefs=c(b_te, b_ze, b_tx, b_zx, b_zx_star)
, sigma_epsilon_ij  = sigma_epsilon_ij, mu0=mu0,
                               coefsE=c(ef.main,ef.main.sq, ef.main.ran.rows),
                               coefsT=c(ef.main,ef.main.sq, ef.main.ran.columns))
  obj <- make_obj_for_traitenv(Y,E,T)
  glm_4th <- pmax_PermutationTest(obj, test_statistics_TE, nrepet = nrepet)
  pvals[i,]=c(glm_4th["glm",-1],glm_4th["fourth",-1])
}
#warnings() # glm.fit: fitted rates numerically 0 occurred
rejection_rate_llm = colMeans(1*(pvals  <= nominal_level)) #llm = log-linear model
(threshold <- (nominal_level + 2*sqrt(nominal_level*(1-nominal_level)/n_simul)))
suspect.rej<- rejection_rate_llm  > threshold
# glm_prow glm_pcol glm_pmax 4th_prow 4th_pcol 4th_pmax
# 0.159    0.091    0.061    0.161    0.091    0.059
# pmax of both glm and fourth corner ~ 0.06 that is:
# slightly inflated with this smaller number of sites and species, although not sig
nificantly so
print(rejection_rate_llm)
print(suspect.rej)
# glm_prow glm_pcol glm_pmax 4th_prow 4th_pcol 4th_pmax
# TRUE     FALSE     FALSE     TRUE     FALSE     FALSE
#

# Section 2c log-linear simulation model b_tx != 0 10 random traits----------------
-----------------------------

mu0 <- 30
# effect sizes in log-linear model (b1,b2,b2 = b_te, b_ze, b_tx)
# with x and z random nuisance variables.
b_te <- 0   # the null model, without t-e interaction
b_ze <- 0.6
b_tx <- 0.6
b_zx <- 0
b_zx_star <- 0.2
sigma_epsilon_ij <- 0.2 # min(0.1, b_te + b_ze + b_tx)   # unstructured interaction


ef.main <- 0.05  # runif(1,min=0.1, max = ef.main)
ef.main.sq <-  -0.1
ef.main.ran.rows <- 0.1
```

```
ef.main.ran.columns <- 0.1

if (ef.main.sq < 0){
  opt <-  -ef.main/(2*ef.main.sq)
  tol <- 1/sqrt(-2*ef.main.sq)
}
print(opt); print(tol)

pvals <- matrix(NA, nrow = n_simul, ncol = 6);
colnames(pvals)=c( "glm_prow", "glm_pcol", "glm_pmax","4th_prow", "4th_pcol", "4th_
pmax")

for (i in 1:n_simul){
  Tlist <- generate_Tdata(n_species, n_traits, cor_Tdat, b = rep(1,n_traits))
  Tdat <- Tlist$Tdat; T <-Tlist$T; c.true <-Tlist$coefficients
  E <- as.matrix(rnorm(n_communities))
  Y <- generate_RC_community(E= E, T= T, coefs=c(b_te, b_ze, b_tx, b_zx, b_zx_star)
, sigma_epsilon_ij  = sigma_epsilon_ij, mu0=mu0,
                             coefsE=c(ef.main,ef.main.sq, ef.main.ran.rows),
                             coefsT=c(ef.main,ef.main.sq, ef.main.ran.columns))
  obj <- make_obj_for_traitenv(Y,E,Tdat)
  glm_4th <- pmax_PermutationTest(obj, test_statistics_TE, nrepet = nrepet)
  pvals[i,]=c(glm_4th["glm",-1],glm_4th["fourth",-1])
}
#warnings() # glm.fit: fitted rates numerically 0 occurred
rejection_rate_llm = colMeans(1*(pvals  <= nominal_level)) #llm = log-linear model
(threshold <- (nominal_level + 2*sqrt(nominal_level*(1-nominal_level)/n_simul)))
suspect.rej<- rejection_rate_llm  > threshold
# glm_prow glm_pcol glm_pmax 4th_prow 4th_pcol 4th_pmax
# 0.769    0.028    0.020    0.531    0.055    0.046
# pmax of both glm and fourth corner <0.05 that is: not inflated in this multi-trai
t setting
print(rejection_rate_llm)
print(suspect.rej)
}
```

2.4 Results log of example R script

The purpose of Sections 1a (10 random traits) was to show that `anova.traitglm` can give very high type I error: the type I error is 0.9 instead of <0.05. In sections 1b and 2a (using a single trait), the error 0.3 instead of <0.05. In the remaining sections the bad behavior of `anova.traitglm` is taken for granted and the focus on the type I error control of the proposed solution, the p_{max} test. Here the number of simulations is 1000. With the lower number of species and sites, the type I error is only slightly inflated (type I error of 0.06) in section 2b and, with 10 random traits, no inflation.

```
rm(list=ls(all=TRUE))  # remove all existing items from the workspace
library(mvabund)
source("generate_trait_env_community_data.r")
source("te_test_functions.r")


# Section 1a Gaussian response simulations with 10 random traits ------------------
```

```
--------------------------------
# n = 20; m = 11; q = 10
n_communities <- 20 #n
n_species <- 11 # m
mu_max <- 10
n_traits <- 10  #q
cor_Tdat <- 0


n_simul <-  10  # in the paper we did 1000 unless otherwise noted.
nrepet <- 39
nominal_level <-  0.05
(threshold <- (nominal_level + 2*sqrt(nominal_level*(1-nominal_level)/n_simul)))

## [1] 0.1878405

# Gaussian response model settings
mu_max <- 10; tolerance = 2
rho_T = 0; rho_E = 1 # Eqn 1 and Eqn A.1/A.2

set.seed(1257)

pvals <- matrix(NA, nrow = n_simul, ncol = 7);
colnames(pvals)=c("anova.traitglm", "glm_prow", "glm_pcol", "glm_pmax","4th_prow",
"4th_pcol", "4th_pmax")

for (i in 1:n_simul){
    Tlist <- generate_Tdata(n_species, n_traits, cor_Tdat, b = rep(1,n_traits))
    Tdat <- Tlist$Tdat; T <-Tlist$T; c.true <-Tlist$coefficients
    E <- as.matrix(rnorm(n_communities))
    z <- rnorm(n_species); x <- rnorm(n_communities)
    z_star <- rho_T * T + sqrt(1 - rho_T^2)*z # rho_T = 0 == trait random; Eqn A.2
    x_star <- rho_E * E + sqrt(1 - rho_E^2)*x # rho_E = 1 == env NOT random  Eqn A.
2
    # thus: as rho_T = 0 and rho_E = 1
    # z_star = z # the trait governing the Gaussian model, but, alas, unobserved (l
atent)
    # x_star = E # the observed trait that is also the one governing the Gaussian m
odel
    Y <- generate_community_Gaussian_response(tolerance = tolerance, E = x_star, T
= z_star, mu_max = mu_max)
    obj <- make_obj_for_traitenv(Y,E,Tdat)
    res_anova.traitglm <- anova_traitglm(obj, nrepet = nrepet)
    res_glm_pmax <- pmax_PermutationTest(obj, trait_env_glm_LR, nrepet = nrepet)
    res_4th_pmax <- pmax_PermutationTest(obj, fourthcorner, nrepet = nrepet, withgl
m = FALSE)
    pvals[i,]= c(res_anova.traitglm["pval"], res_glm_pmax[-1],res_4th_pmax[-1])
}

## Using block resampling...
## Using block resampling...
## Using block resampling...
## Using block resampling...

## Warning: glm.fit: fitted rates numerically 0 occurred

## [similar messages and warnings deleted]

## Warning: glm.fit: fitted rates numerically 0 occurred

#warnings() # glm.fit: fitted rates numerically 0 occurred
rejection_rate_q = colMeans(1*(pvals  <= nominal_level))
suspect.rej<- rejection_rate_q  > threshold
print(rejection_rate_q)

## anova.traitglm       glm_prow       glm_pcol       glm_pmax       4th_prow
##            0.9            0.7            0.0            0.0            0.6
```

```
##       4th_pcol       4th_pmax
##            0.1            0.1

# # anova.traitglm       glm_prow       glm_pcol       glm_pmax       4th_prow
# # 0.9            0.7            0.0            0.0            0.6
# # 4th_pcol       4th_pmax
# # 0.1            0.1

print(suspect.rej)

## anova.traitglm       glm_prow       glm_pcol       glm_pmax       4th_prow
##           TRUE           TRUE          FALSE          FALSE           TRUE
##       4th_pcol       4th_pmax
##          FALSE          FALSE

# # anova.traitglm       glm_prow       glm_pcol       glm_pmax       4th_prow
# # TRUE             TRUE          FALSE          FALSE            TRUE
# # 4th_pcol       4th_pmax
# # FALSE          FALSE
# # Conclusion 1: anova.traitglm gives highly elevated type I error (detectable eve
n with n_simul = 10!)
# # Conclusion 2: glm_pmax gives controls the type I error rate
# # Conclusion 3: 4th_pmax gives controls the type I error rate
# # Remark: Inspection of pvals shows that in this many traits case,
# # glm and 4th (actually the RLQ intertia) differ on a per data set basis;
# # this is not only due different in the permutation, but is generally expected,
# # particularly so when cor_Tdat!=0 or the linear combination b does not simply sp
ecify a sum

# Section 1b Gaussian response simulations with one random trait ------------------
--------------------------------

pvals <- matrix(NA, nrow = n_simul, ncol = 7);
colnames(pvals)=c("anova.traitglm", "glm_prow", "glm_pcol", "glm_pmax","4th_prow",
"4th_pcol", "4th_pmax")

for (i in 1:n_simul){
  T <- as.matrix(rnorm(n_species))
  E <- as.matrix(rnorm(n_communities))
  z <- rnorm(n_species); # x <- rnorm(n_communities)
  z_star <- rho_T * T + sqrt(1 - rho_T^2)*z # rho_T = 0 == trait random; Eqn A.2
  x_star <- rho_E * E + sqrt(1 - rho_E^2)*x # rho_E = 1 == env NOT random  Eqn A.2
  # thus: if rho_T = 0 and rho_E = 1
  # z_star = z # the trait governing the Gaussian model, but, alas, unobserved (lat
ent)
  # x_star = E # the observed trait that is also the one governing the Gaussian mod
el
  Y <- generate_community_Gaussian_response(tolerance = tolerance, E = x_star, T =
z_star, mu_max = mu_max)
  obj <- make_obj_for_traitenv(Y,E,T)
  res_anova.traitglm <- anova_traitglm(obj, nrepet = nrepet)
# # in the next three lines, independent row and columns permutations are used for
the two tests
#   res_glm_pmax <- pmax_PermutationTest(obj, trait_env_glm_LR, nrepet = nrepet)
#   res_4th_pmax <- pmax_PermutationTest(obj, fourthcorner, nrepet = nrepet)
#   pvals[i,]= c(res_anova.traitglm["pval"], res_glm_pmax[-1],res_4th_pmax[-1])
# # in the next line below, the same row and columns permutations are used for the
two tests
# # so as to show that they give about the same result if n_env=n_traits=1
  glm_4th <- pmax_PermutationTest(obj, test_statistics_TE, nrepet = nrepet)
  pvals[i,]=c(res_anova.traitglm["pval"],glm_4th["glm",-1],glm_4th["fourth",-1])
}

## Using block resampling...
## [similar messages deleted]
```

```
## Using block resampling...

#warnings() # glm.fit: fitted rates numerically 0 occurred
rejection_rate_1 = colMeans(1*(pvals  <= nominal_level))
suspect.rej<- rejection_rate_1  > threshold
print(rejection_rate_1)

## anova.traitglm       glm_prow       glm_pcol       glm_pmax       4th_prow
##            0.3            0.3            0.0            0.0            0.3
##       4th_pcol       4th_pmax
##            0.0            0.0

# anova.traitglm       glm_prow       glm_pcol       glm_pmax       4th_prow
# 0.3            0.3            0.0            0.0            0.3
# 4th_pcol       4th_pmax
# 0.0            0.0
print(suspect.rej)

## anova.traitglm       glm_prow       glm_pcol       glm_pmax       4th_prow
##           TRUE           TRUE          FALSE          FALSE           TRUE
##       4th_pcol       4th_pmax
##          FALSE          FALSE

# # anova.traitglm       glm_prow       glm_pcol       glm_pmax       4th_prow
# # TRUE           TRUE          FALSE          FALSE           TRUE
# # 4th_pcol       4th_pmax
# # FALSE          FALSE
# # Conclusion 1: anova.traitglm gives elevated type I error (detectable even with
n_simul = 10!)
# # Conclusion 2: glm_pmax gives controls the type I error rate
# # Conclusion 3: 4th_pmax gives controls the type I error rate
# # Remark: Inspection of pvals shows that in this one-one case,
# # glm and 4th do hardly differ on a per data set basis
pvals[, 1+(1:3)]- pvals[,4+(1:3)]

##       glm_prow glm_pcol glm_pmax
##  [1,]    0.000        0        0
##  [2,]    0.000        0        0
##  [3,]    0.025        0        0
##  [4,]    0.000        0        0
##  [5,]    0.000        0        0
##  [6,]    0.000        0        0
##  [7,]    0.000        0        0
##  [8,]    0.000        0        0
##  [9,]    0.000        0        0
## [10,]    0.000        0        0

# # summary:
id = apply(abs(pvals[, 1+(1:3)]- pvals[,4+(1:3)]) > 0, 1, sum)
sum(id);  # unequal in one data set

## [1] 1

which(id >0) # namely the seven-th data set, if which(id >0) = 7

## [1] 3

pvals[id,]

## anova.traitglm       glm_prow       glm_pcol       glm_pmax       4th_prow
##          0.325          0.175          0.350          0.350          0.175
##       4th_pcol       4th_pmax
##          0.350          0.350

rm(z, z_star, x_star, Y, E, T)

# Section 2a log-linear simulation model b_tx = 0 ---------------------------------
```

```
------------

mu0 = 30
# effect sizes in log-linear model (b1,b2,b2 = b_te, b_ze, b_tx)
# with x and z random nuisance variables.
b_te <- 0   # the null model, without t-e interaction
b_ze <- 0.8
b_tx <- 0
b_zx <- 0
b_zx_star <- 0.2
sigma_epsilon_ij <- 0.2 # min(0.1, b_te + b_ze + b_tx)   # unstructured interaction


ef.main <- 0.05  # runif(1,min=0.1, max = ef.main)
ef.main.sq <-  -0.1
ef.main.ran.rows <- 0.1
ef.main.ran.columns <- 0.1

if (ef.main.sq < 0){
  opt <-  -ef.main/(2*ef.main.sq)
  tol <- 1/sqrt(-2*ef.main.sq)
}
print(opt); print(tol)

## [1] 0.25

## [1] 2.236068

pvals <- matrix(NA, nrow = n_simul, ncol = 7);
colnames(pvals)=c("anova.traitglm", "glm_prow", "glm_pcol", "glm_pmax","4th_prow",
"4th_pcol", "4th_pmax")

for (i in 1:n_simul){
  T <- as.matrix(rnorm(n_species))
  E <- as.matrix(rnorm(n_communities))
  Y <- generate_RC_community(E= E, T= T, coefs=c(b_te, b_ze, b_tx, b_zx, b_zx_star)
, sigma_epsilon_ij  = sigma_epsilon_ij, mu0=mu0,
                                  coefsE=c(ef.main,ef.main.sq, ef.main.ran.rows),
                                  coefsT=c(ef.main,ef.main.sq, ef.main.ran.columns))
  obj <- make_obj_for_traitenv(Y,E,T)
  res_anova.traitglm <- anova_traitglm(obj, nrepet = nrepet)
  glm_4th <- pmax_PermutationTest(obj, test_statistics_TE, nrepet = nrepet)
  pvals[i,]=c(res_anova.traitglm["pval"],glm_4th["glm",-1],glm_4th["fourth",-1])
}

## Using block resampling...
## [similar messages deleted]
## Using block resampling...

#warnings() # glm.fit: fitted rates numerically 0 occurred
rejection_rate_llm = colMeans(1*(pvals  <= nominal_level)) #llm = log-linear model
suspect.rej<- rejection_rate_llm  > threshold
print(rejection_rate_llm)

## anova.traitglm       glm_prow       glm_pcol       glm_pmax       4th_prow
##            0.3            0.2            0.0            0.0            0.2
##       4th_pcol       4th_pmax
##            0.0            0.0

# anova.traitglm       glm_prow       glm_pcol       glm_pmax       4th_prow
# 0.3            0.2            0.0            0.0            0.2
# 4th_pcol       4th_pmax
# 0.0            0.0
print(suspect.rej)

## anova.traitglm       glm_prow       glm_pcol       glm_pmax       4th_prow
##           TRUE           TRUE          FALSE          FALSE           TRUE
```

```
##       4th_pcol       4th_pmax
##          FALSE          FALSE

# anova.traitglm       glm_prow       glm_pcol       glm_pmax       4th_prow
#       TRUE           TRUE          FALSE          FALSE           TRUE
# 4th_pcol       4th_pmax
# FALSE          FALSE
# Conclusion 1: anova.traitglm gives elevated type I error (detectable even with n_
simul = 10!)
# Conclusion 2: glm_pmax gives controls the type I error rate
# Conclusion 3: 4th_pmax gives controls the type I error rate
# Remark: Inspection of pvals shows that in this one-one case,
# glm and 4th do differ slightly on a per data set basis
pvals[, 1+(1:3)]- pvals[,4+(1:3)]

##       glm_prow glm_pcol glm_pmax
##  [1,]    0.000    0.000    0.000
##  [2,]   -0.025   -0.025   -0.025
##  [3,]    0.000    0.000    0.000
##  [4,]    0.000    0.000    0.000
##  [5,]    0.000    0.000    0.000
##  [6,]    0.000    0.000    0.000
##  [7,]    0.000    0.000    0.000
##  [8,]    0.000    0.000    0.000
##  [9,]    0.000    0.000    0.000
## [10,]    0.000    0.000    0.000

# # summary:
id = apply(abs(pvals[, 1+(1:3)]- pvals[,4+(1:3)]) > 0, 1, sum)
sum(id);  # unequal in ... data sets

## [1] 3

which(id >0) # namely the seven-th data set, if which(id >0) = 7

## [1] 2

#pvals[id,]


# Section 2b log-linear simulation model b_tx != 0 single trait--------------------
-------------------------

n_simul <- 1000
# simulations with anova.traitglm as it was already shown to be wrong in this kind
of model
# and it takes far too long with 1000 simulations...
mu0 <- 30
# effect sizes in log-linear model (b1,b2,b2 = b_te, b_ze, b_tx)
# with x and z random nuisance variables.
b_te <- 0   # the null model, without t-e interaction
b_ze <- 0.6
b_tx <- 0.6
b_zx <- 0
b_zx_star <- 0.2
sigma_epsilon_ij <- 0.2 # min(0.1, b_te + b_ze + b_tx)   # unstructured interaction


ef.main <- 0.05  # runif(1,min=0.1, max = ef.main)
ef.main.sq <-  -0.1
ef.main.ran.rows <- 0.1
ef.main.ran.columns <- 0.1

if (ef.main.sq < 0){
  opt <-  -ef.main/(2*ef.main.sq)
  tol <- 1/sqrt(-2*ef.main.sq)
```

```
}
print(opt); print(tol)

## [1] 0.25

## [1] 2.236068

pvals <- matrix(NA, nrow = n_simul, ncol = 6);
colnames(pvals)=c( "glm_prow", "glm_pcol", "glm_pmax","4th_prow", "4th_pcol", "4th_
pmax")

for (i in 1:n_simul){
  T <- as.matrix(rnorm(n_species))
  E <- as.matrix(rnorm(n_communities))
  Y <- generate_RC_community(E= E, T= T, coefs=c(b_te, b_ze, b_tx, b_zx, b_zx_star)
, sigma_epsilon_ij  = sigma_epsilon_ij, mu0=mu0,
                             coefsE=c(ef.main,ef.main.sq, ef.main.ran.rows),
                             coefsT=c(ef.main,ef.main.sq, ef.main.ran.columns))
  obj <- make_obj_for_traitenv(Y,E,T)
  glm_4th <- pmax_PermutationTest(obj, test_statistics_TE, nrepet = nrepet)
  pvals[i,]=c(glm_4th["glm",-1],glm_4th["fourth",-1])
}
#warnings() # glm.fit: fitted rates numerically 0 occurred
rejection_rate_llm = colMeans(1*(pvals  <= nominal_level)) #llm = log-linear model
(threshold <- (nominal_level + 2*sqrt(nominal_level*(1-nominal_level)/n_simul)))

## [1] 0.06378405

suspect.rej<- rejection_rate_llm  > threshold
# glm_prow glm_pcol glm_pmax 4th_prow 4th_pcol 4th_pmax
# 0.159    0.091    0.061    0.161    0.091    0.059
# pmax of both glm and fourth corner ~ 0.06 that is:
# slightly inflated with this smaller number of sites and species, although not sig
nificantly so
print(rejection_rate_llm)

## glm_prow glm_pcol glm_pmax 4th_prow 4th_pcol 4th_pmax
##    0.159    0.091    0.061    0.161    0.091    0.059

print(suspect.rej)

## glm_prow glm_pcol glm_pmax 4th_prow 4th_pcol 4th_pmax
##     TRUE     TRUE    FALSE     TRUE     TRUE    FALSE

# glm_prow glm_pcol glm_pmax 4th_prow 4th_pcol 4th_pmax
# TRUE    FALSE    FALSE     TRUE    FALSE    FALSE
#

# Section 2c log-linear simulation model b_tx != 0 10 traits-----------------------
----------------------

mu0 <- 30
# effect sizes in log-linear model (b1,b2,b2 = b_te, b_ze, b_tx)
# with x and z random nuisance variables.
b_te <- 0   # the null model, without t-e interaction
b_ze <- 0.6
b_tx <- 0.6
b_zx <- 0
b_zx_star <- 0.2
sigma_epsilon_ij <- 0.2 # min(0.1, b_te + b_ze + b_tx)   # unstructured interaction


ef.main <- 0.05  # runif(1,min=0.1, max = ef.main)
ef.main.sq <-  -0.1
ef.main.ran.rows <- 0.1
ef.main.ran.columns <- 0.1
```

```
if (ef.main.sq < 0){
  opt <-  -ef.main/(2*ef.main.sq)
  tol <- 1/sqrt(-2*ef.main.sq)
}
print(opt); print(tol)

## [1] 0.25

## [1] 2.236068

pvals <- matrix(NA, nrow = n_simul, ncol = 6);
colnames(pvals)=c( "glm_prow", "glm_pcol", "glm_pmax","4th_prow", "4th_pcol", "4th_
pmax")

for (i in 1:n_simul){
  Tlist <- generate_Tdata(n_species, n_traits, cor_Tdat, b = rep(1,n_traits))
  Tdat <- Tlist$Tdat; T <-Tlist$T; c.true <-Tlist$coefficients
  E <- as.matrix(rnorm(n_communities))
  Y <- generate_RC_community(E= E, T= T, coefs=c(b_te, b_ze, b_tx, b_zx, b_zx_star)
, sigma_epsilon_ij  = sigma_epsilon_ij, mu0=mu0,
                              coefsE=c(ef.main,ef.main.sq, ef.main.ran.rows),
                              coefsT=c(ef.main,ef.main.sq, ef.main.ran.columns))
  obj <- make_obj_for_traitenv(Y,E,Tdat)
  glm_4th <- pmax_PermutationTest(obj, test_statistics_TE, nrepet = nrepet)
  pvals[i,]=c(glm_4th["glm",-1],glm_4th["fourth",-1])
}
#warnings() # glm.fit: fitted rates numerically 0 occurred
rejection_rate_llm = colMeans(1*(pvals  <= nominal_level)) #llm = log-linear model
(threshold <- (nominal_level + 2*sqrt(nominal_level*(1-nominal_level)/n_simul)))

## [1] 0.06378405

suspect.rej<- rejection_rate_llm  > threshold
# glm_prow glm_pcol glm_pmax 4th_prow 4th_pcol 4th_pmax
# 0.769    0.028    0.020    0.531    0.055    0.046
# pmax of both glm and fourth corner <0.05 that is: not inflated in this multi-trai
t setting
print(rejection_rate_llm)

## glm_prow glm_pcol glm_pmax 4th_prow 4th_pcol 4th_pmax
##    0.769    0.028    0.020    0.531    0.055    0.046

print(suspect.rej)

## glm_prow glm_pcol glm_pmax 4th_prow 4th_pcol 4th_pmax
##     TRUE    FALSE    FALSE     TRUE    FALSE    FALSE
```

References

Dray, S., P. Choler, S. Dolédec, P. R. Peres-Neto, W. Thuiller, S. Pavoine, and C. J. F. ter Braak. 2014. Combining the fourth-corner and the RLQ methods for assessing trait responses to environmental variation. Ecology 95:14-21.

Jamil, T., W. A. Ozinga, M. Kleyer, and C. J. F. ter Braak. 2013. Selecting traits that explain species–environment relationships: a generalized linear mixed model approach. Journal of Vegetation Science 24:988-1000.
