# Supplementary material for: A critical issue in model-based inference for studying trait-based community assembly and a solution
Source: PeerJ. 2017 Jan 12;5:e2885. doi: 10.7717/peerj.2885 (PMC5237366; doi:10.7717/peerj.2885)
Supplement: Appendix S3 [file peerj-05-2885-s003.pdf]

A critical issue in model-based inference for studying trait-based community assembly and a solution

Cajo J. F. Ter Braak, Pedro R. Peres-Neto and Stéphane Dray

Appendix S3

3. Why does site-based statistical testing in a GLM fail?

The reason of the failure of the test is that the importance of **e** in the species-by-sites interaction is not a sufficient condition for trait-environment association, even when the test-statistic tries to capture the **t**-**e** interaction only. Consider the GLMM model

$$\log(\mu_{ij}) = R_i + C_j + \beta_j e_i \text{ with } \beta_j = b_0 + b_{te} t_j + \varepsilon_{\beta j} \quad \text{(A.7)}$$

with β_j a species-specific slope with respect to **e**, modelled as a linear model of trait **t**, with intercept b_0 and slope b_{te}, and with $\varepsilon_{\beta j}$ a normally distributed error term with mean 0 and variance σ_β^2. By inserting the model for β_j in the log-linear model, we see that b_{te} is indeed the coefficient of the interaction $t_j e_i$. The term $b_0 e_i$ can be absorbed in the row main effect R_i, and $\varepsilon_{\beta j} e_i$ represents additional species-specific random variation that interacts with the observed environment. For judging the significance of b_{te} one can plot estimates of β_j against t_j and add the fitted line to the plot, as Jamil et al. (2013) did in their Fig. 3. Clearly, the scatter of the species-specific slopes around the fitted line gives an insight about the importance of the relationship. This scatter, in Equation (2) represented by $\varepsilon_{\beta j}$, is taken into account in the GLMM model, but not in the GLM model with site-based resampling as in anova.traitglm. Therefore, resampling of sites, as in anova.traitglm, is expected to generate inflated type I error rates, unless the parameter $\sigma_\beta = 0$.
