# Supplementary figures and images for: A critical issue in model-based inference for studying trait-based community assembly and a solution
Source: PeerJ. 2017 Jan 12;5:e2885. doi: 10.7717/peerj.2885 (PMC5237366; doi:10.7717/peerj.2885)

# Residuals vs Fitted

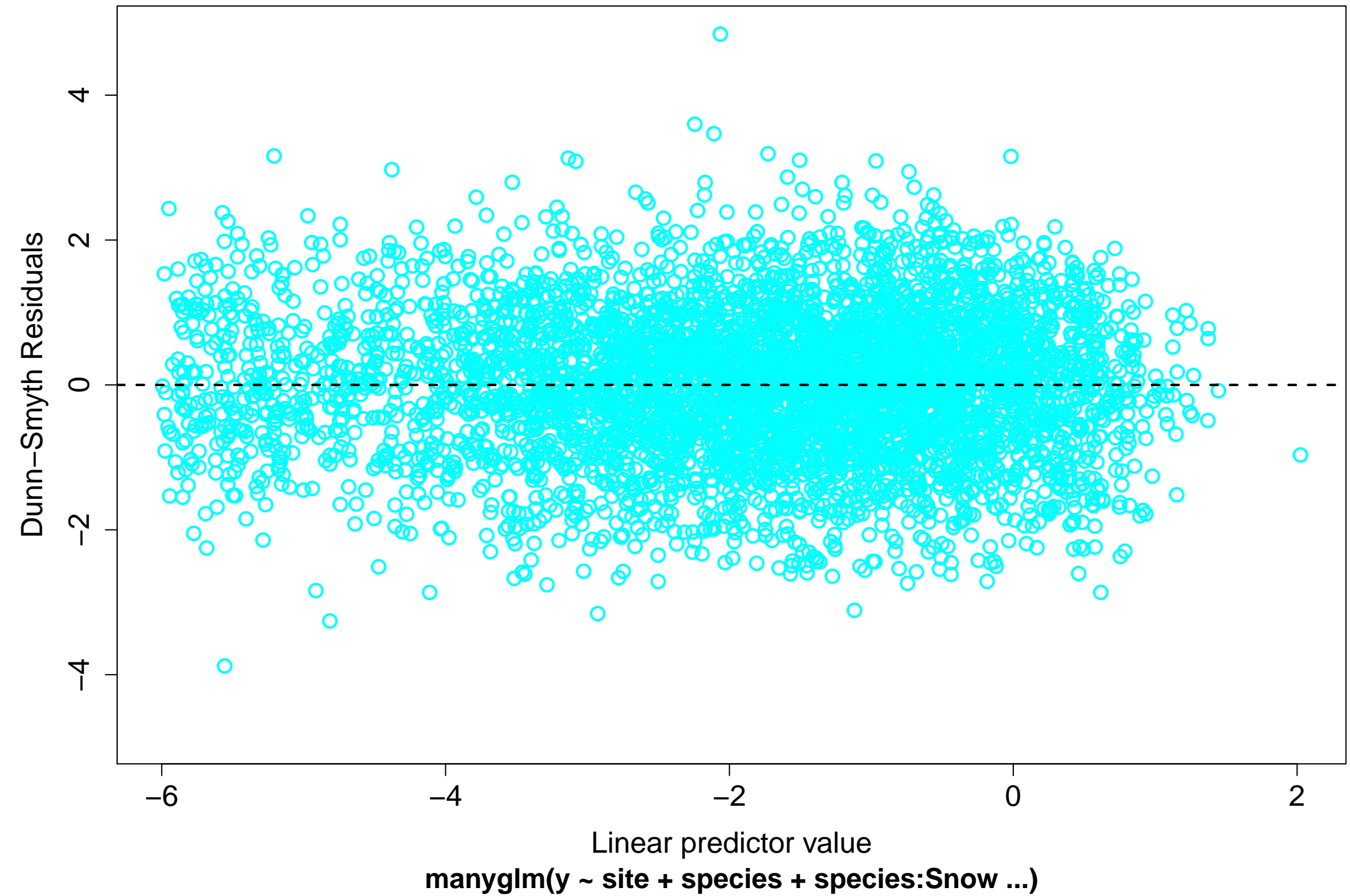

Supplement: Figure S1 — Dunn-Smith residuals of the model ‘site+species+species:Snow’ against the fitted values in the aravo data set. [file peerj-05-2885-s004.pdf]
